# Morphological and molecular identification of the brown dog tick *Rhipicephalus sanguineus* and the camel tick *Hyalomma dromedarii* (Acari: Ixodidae) vectors of Rickettsioses in Egypt

**DOI:** 10.14202/vetworld.2016.1087-1101

**Published:** 2016-10-18

**Authors:** Hend H. A. M. Abdullah, Amal El-Molla, Fayez A. Salib, Nesreen A. T. Allam, Alaa A. Ghazy, Sobhy Abdel-Shafy

**Affiliations:** 1 Department of Parasitology and Animal Diseases, Division of Veterinary Research, National Research Centre, Dokki, Giza, Egypt; 2 Department of Infectious Diseases, Faculty of Veterinary Medicine, Cairo University, Giza, Egypt

**Keywords:** hard ticks, light microscope, phylogenetic analysis, polymerase chain reaction, Rickettsiae, scanning electron microscope

## Abstract

**Aim:**

Rickettsioses have an epidemiological importance that includes pathogens, vectors, and hosts. The dog tick *Rhipicephalus sanguineus* and the camel tick *Hyalomma dromedarii* play important roles as vectors and reservoirs of Rickettsiae. The aim of this study was to determine the prevalence of Rickettsiae in ixodid ticks species infesting dogs and camels in Egypt, in addition to, the morphological and molecular identification of *R. sanguineus* and *H. dromedarii*.

**Materials and Methods:**

A total of 601 and 104 of ticks’ specimens were collected from dogs and camels, respectively, in Cairo, Giza and Sinai provinces. Hemolymph staining technique and *OmpA* and *gltA* genes amplification were performed to estimate the prevalence rate of Rickettsiae in ticks. For morphological identification of tick species, light microscope (LM) and scanning electron microscope (SEM) were used. In addition to the phylogenetic analyses of 18S rDNA, Second internal transcript spacer, 12S rDNA, cytochrome c oxidase subunit-1, and 16S rDNA were performed for molecular identification of two tick species.

**Results:**

The prevalence rate of Rickettsiae in ticks was 11.6% using hemolymph staining technique and 6.17% by *OmpA* and *gltA* genes amplification. Morphological identification revealed that 100% of dogs were infested by *R. sanguineus* while 91.9% of camels had been infested by *H. dromedarii*. The phylogenetic analyses of five DNA markers confirmed morphological identification by LM and SEM. The two tick species sequences analyses proved 96-100% sequences identities when compared with the reference data in Genbank records.

**Conclusion:**

The present studies confirm the suitability of mitochondrial DNA markers for reliable identification of ticks at both intra- and inter-species level over the nuclear ones. In addition to, the detection of Rickettsiae in both ticks’ species and establishment of the phylogenetic status of *R. sanguineus* and *H. dromedarii* would be useful in understanding the epidemiology of ticks and tick borne rickettsioses in Egypt.

## Introduction

Since 1960s, multiple traditional smear staining, serological, immunohistochemical as well as molecular studies had indicated that vectors, rodents, animals as well as human in Egypt are endemic with rickettsial agents [1-6]. Rickettsioses are recently worldwide recognized as emerging and reemerging arthropods-borne infectious diseases with zoonotic importance [7-13]. Bacteria of the order Rickettsiales were first described as obligatory intracellular short Gram-negative bacillary microorganisms that retained basic fuchsin when stained by the method of Gimenez [14-16]. Despite its unpretentious, the hemolymph staining keeps ticks undamaged and the infected specimens can be used in further identification and/or isolation techniques of Rickettsiae [14]. Ticks are the main vectors of Rickettsiae, especially spotted fever group (SFG) that was transmitted through transstadial and transovarial transmission [17-19]. During the last decade, the taxonomy of Rickettsiae has undergone extensive reorganization [17,20]. Up to date, the order Rickettsiales includes two families; Anaplasmataceae and Rickettsiaceae, which necessitated advanced genetic guidelines for the reclassification of rickettsial isolates at the genus, group, and species levels, utilizing the multilocus sequencing of five genes including 16S ribosomal ribonucleic acid (16S rRNA), *gltA*, *ompA*, *ompB*, and *sac*4 genes [21,22]. In the last years, the diagnosis of *Rickettsia* spp. was depending on the molecular tools. The outer membrane protein A of the cell surface antigen (*OmpA*) and citrate synthase (*gltA*) genes is only present in SFG Rickettsiae [23-26]. These two genes have high discrimination power to detection of *Rickettsia* spp. due to high variability within SFG [23,27].

In Egypt, *Rhipicephalus sanguineus* is the main dog infesting ticks [28-30]. While the most common ixodid ticks (order Parasitiformes of subclass Acari) infesting camels are *Hyalomma* species, especially *Hyalomma dromedarii, Hyalomma marginatum, Hyalomma excavatum*, and *Hyalomma impeltatum* [6,31,32]. Being hematophagous of biohazards danger and public health importance, they have been accused participant in emerging and/or reemerging tick-borne human and animal infectious diseases such as SFG, lyme disease, babesiosis; Q-fever and life-threatening arboviruses [6,17,33-36] through their two or three host life cycle [28,29]. The accurate taxonomy of tick species is a very important to control tick-borne diseases. The morphological identification using light microscope (LM) clarifies mainly the size and color of tick species besides other obvious characteristics such as mouth parts, the outline of body and scutum. However, scanning electron microscope (SEM) describes the fine characteristics which unclear by LM such as genital opening, punctuations, spiracles, and grooves [37]. Traditionally, taxonomical identification of ticks was based on the morphological criteria of adult ticks through LM or SEM, nonetheless, the applicability of this method is difficult when the specimens are engorged with blood, physically damaged, in immature stages; i.e. eggs, larvae, or nymphs [38,39] and very doubtful at subspecies and/or one group level; *R. sanguineus* group [40-46].

In recent years, the molecular characterization for taxonomic identification and phylogenetic purposes of ticks has been indispensable by DNA markers, including nuclear (18S rRNA) and mitochondrial (12S rRNA and 16S rRNA, and cytochrome c oxidase subunit-1 [CO1]) genes and nuclear regulatory non-translated stretches (second internal transcribed spacer [ITS2]) [44,47-50]. The 18S rRNA is best used for genera level identification [51] while the 16S rRNA, CO1, and ITS2 are the most useful markers for ticks taxonomy at species level [39,44,47-50,52-56]. The 12S rRNA is used as a tool for examining relationships among recently diverged branches of tick phylogenies [50,53,57].

In this study, the objectives were the determination of the prevalence of ticks-borne Rickettsiae with regard to ixodid ticks’ species infestation in dogs and camels in Cairo, Giza and Sinai provinces in Egypt. In addition, to assess the ability of five DNA markers to confirm the identity of the most common tick species *R. sanguineus* and *H. dromedarii* at both species and genera levels besides the phylogenetic relationship between Egyptian tick species and others worldwide by sequences alignment with GenBank related records.

## Materials and Methods

### Ethical approval

Ethical approval of the study was obtained from the Ethics Committee of National Research Centre, Giza, Egypt.

### Animals

A total of 261 animals were investigated for tick infestation. The inspected animals were dogs that were collected from Cairo province and camels which were collected from Cairo, Giza, and Sinai provinces. These animals were examined for the presence of ticks in neck, chest area, and around the soft parts of their bodies such as the inner sides of the hind and forelegs, perineal area, inner surface of ears and udders [58].

### Ticks

A total of 705 adult ticks were collected (601 ticks from dogs and 104 ticks from camels) from Cairo, Giza, and Sinai provinces in Egypt. In addition, 9 nymphs were included in tick specimens. Ixodid ticks were collected during the period from March 2012 to October 2014. Ticks were detached from animals using strong forceps into plastic tubes covered by a piece of cloth and secured by rubber band. The field data of each sample such as date, locality, and number of examined animals were recorded [32]. Ticks were brought alive to the laboratory for further identification and investigation.

### Microscopical detection of Rickettsiae using hemolymph staining smears

All collected ticks were examined for Rickettsiae using hemolymph staining technique. According to Burgdorfer [59], hemolymph was impressed on slide following scissors amputating the distal portion of legs, fixed by air dry then stained with Gimenez stain [14]. The prepared hemolymph slides were examined under oil emersion lens using ordinary microscope (Zeiss).

### Molecular identification of ticks-borne Rickettsiae by *OmpA* and *gltA* genes amplification

Polymerase chain reactions (PCRs) were performed to specifically amplify the *Rickettsia* spp. *OmpA* and *gltA* genes sequence by the appropriate primer set designed according to Roux *et al*. [24], Fournier *et al*. [25], Mediannikov *et al*. [26] (Table-1).*OmpA* primers, both the forward 190.70-F and the reverse 190.701-R primers, were designed to span the nucleotides positions from 70 to 90 and 701 to 681, respectively, as the predicted product size are ranged from 590 to 634 bp [60]. Moreover, primers CS2d-F and CSEnd-R were amplified the full-length of the *gltA* gene, as the predicted product size are ranged from 852 to 1265 bp, therefore; CS2d primer was designed to be completely homologous to the corresponding portion of the gene of *Rickettsia conorii* for only SFG [26].

**Table-1:** Primers utilized in amplification and sequencing of genes.

DNA marker	5’- primers sequences-3’	Amplified fragments	References
*OmpA* gene		590-634 bp	[25]
190.70-F 190.701-R	5’-ATGGCGAATATTTCTCCAAAA-3’ 5’-GTTCCGTTAATGGCAGCATCT-3’		
*gltA* gene		852-1265 bp	[24,26]
CS2d-F CSEnd-R	5’-ATGACCAATGAAAATAATAAT-3’ 5’-CTTATACTCTCTATGTACA-3’		
18S rRNA		780 bp	[49]
18S-F 18S-R	5’-CATTAAATCAGTTATGGTTCC-3’ 5’-CGCCGCAATACGAATGC-3’		
ITS2		1200-1600 bp	[50]
ITS2-F ITS2-R	5’-ACATTGCGGCCTTGGGTCTT-3’ 5’-TCGCCTGATCTGAGGTCGAC-3’		
12S rRNA		337-355 bp	[57]
T1B T2A	5’-AAACTAGGATTAGATACCCT-3’ 5’-AATGAGAGCGACGGGCGATGT-3’		
CO1		732-820 bp	[54]
CO1-F CO1-R	5’-GGAACAATATATTTAATTTTTGG-3’ 5’-ATCTATCCCTACTGTAAATATATG-3’		
16SrRNA		455 bp	[49]
16S-F 16S-R	5’-TTAAATTGCTGTRGTATT-3’ 5’-CCGGTCTGAACTCASAWC-3’		

CO1=Cytochrome c oxidase subunit-1, 12S rRNA=12S ribosomal ribonucleic acid, ITS2=Second Internal transcribed spacer

The amplification reactions were performed in a PTC-100™ thermal cycler (MJ Research Inc., USA) under complete aseptic conditions. Each 25 µl total volumes of PCR mixture contained 25-50 ng/µl genomic DNA, 10 pM/µl of each primers, 12.5 µl of ×2 Dream Taq Green PCR master mix (x2 buffer, 0.4 mM deoxynucleotide [dNTP] and 4 mM MgCl_2_; ThermoScientific, California, US), and 9 µl nuclease free water (Qiagen) to complete the total volume of the reactions. All amplifications were performed utilizing the following cycling profile for *OmpA* primers; one cycle at 94°C for 5 min (initial denaturation) followed by 40 cycles consisting of denaturation at 94°C for 1 min, annealing at 59°C for 1 min and elongation at 72°C for 1 min, and the final elongation at 72°C for 10 min, while *gltA* protocol included one cycle at 94°C for 5 min (initial denaturation) followed by 40 cycles of denaturation at 94°C for 1.5 min, annealing at 52°C for 1.5 min and elongation at 72°C for 1.5 min, then the final elongation at 72°C for 20 min [6]. A reagent blank was run simultaneously as a control with every PCR. Amplified products from the PCRs were electrophoresed in 1% agarose gels in Tris-borate-ethylenediaminetetraacetic acid (EDTA) (TBE) buffer (45 mM Tris-borate, 1 mM EDTA, pH 8.3, Sigma-Aldrich) then stained with ethidium bromide (Sigma-Aldrich). A 100 bp ladder (Alliance Bio, USA) was used with each gel. Gels photos were analyzed by Lab Image software (BioRad) [6].

### Morphological identification of tick vectors by LM

The collected ticks were counted and sorted to different genera, species, and sex using dissecting microscope. Tick species were identified morphologically using taxonomic keys of Hoogstraal and Kaiser [28] and Estrada-Pena *et al*. [29]. The two most common tick species (*R. sanguineus* and *H. dromedarii*) were morphologically examined in details using LM, especially the dorsal and ventral surfaces of adult males and females. Male and female ticks were fixed on plasticine one dorsally and another one ventrally. The adult ticks in these two positions were photographed by digital camera fixed on stereomicroscope [32].

### Morphological identification of tick vectors by SEM

*R. sanguineus* and *H. dromedarii* were also examined morphologically in details using SEM. Ticks were prepared for SEM according to the method described by Abdel-Shafy [37]. Ticks individuals were cleaned by water-glycerol-potassium chloride solution, washed in tap water, taken through graded series of alcohol, glued by their dorsal and ventral surfaces to the SEM stub, dried by the dryer (Blazer Union, F1-9496 Blazer/Fürstentun Liechtenstein), mounted on SEM stubs, coated with gold using a S15OA Sputter Coater and examined by SEM.

### Molecular identification of tick vectors by PCR

To confirm the morphological classification proceeded on *R. sanguineus* and *H. dromedarii*, multi-locus sequence typing depending on five tick’s genomic DNA markers amplified fragments was carried out which included; one nuclear gene 18S rRNA gene; and untranslated regulatory sequences of the second ITS2 and three mitochondrial genes 12S rRNA, CO1, and 16S rRNA (Table-1).

#### Primers design

Two pairs of primers were designed that were in accordance to GenBank records of nuclear sequences of the *18S rRNA* gene (18S-F and 18S-R) and the nuclear ribosomal ITS2 (ITS2-F and ITS2-R) depending on previous publications in consequence to this subject [49,50], with predicted products sizes 780 and 1200-1600 bp, respectively (Table-1). Moreover, three pairs of primers were utilized that were complementary to fragments in three different mitochondrial DNA markers (Table-1). They were designed according to the complete mitochondrial genome sequence of *Drosophila yakuba* (NC 001322.1) and modified to be complementary to wide divergent arthropods by aligning mitochondrial genes to DNA GenBank sequences’ records from; *Anopheles gambiae*, *Daphnia pulex*, and *Apismellifera*; genbank accession numbers: NC 002084.1, Z15015, and L06178, respectively. The *12S rRNA* gene primers pair (T1B and T2A) with predicted product sizes approximately 337-355 bp [57]. The CO1 primers pair (CO1-F and CO1-R) was designed according to Chitimia *et al*. [54], while the *16S rRNA* gene primers’ set (16S-F and 16S-R) were designed according to Lv *et al*. [49], with predicted products sizes 732-820 bp and 455 bp, respectively (Table-1).

#### Ticks genomic DNA purification

Total DNA was extracted and purified from the complete body of adult ticks of each tick species after dissection into quarters. Before dissection, each sample was washed in different decreasing concentrations of alcohol (70%, 50%, 30%, 10%) for 1 h per each concentration and finally in double distilled water for 1 h. DNA isolation procedures were performed using high salt concentration protocol [61]. 20 mg from each tick tissues were incubated with lysis buffer (10 mM Tris-HCl, 10 mM EDTA, 3.4 mM SDS and 20 mM NaCl pH 8.0, Sigma-Aldrich), in addition to 1 M DTT (Sigma-Aldrich) and proteinase K (20 mg/ml) (Stratagin) at 56°C overnight, then the supernatant was transferred to new collecting tube (Coaster) to proceed in DNA isolation. Purity and concentration of isolated genomic DNA was measured by nanodrop 2000c (Thermo Scientific). Samples recording <1.5 ratio at 260/280 wavelength were proceeded to second phase of DNA isolation by shaking in phenol/chloroform/isoamylic alcohol (25:24:1, Sigma-Aldrich) and precipitated by adding 2 volumes of ice-cold ethanol for 4-6 h [62]. After centrifugation (at 14,000 rpm for 5 min), the pellet was resuspended in 50 µl Tris-EDTA buffer (10 mM Tris, pH 8.0 and 1 mM EDTA), and the DNA was used as template for PCR and the remaining DNA was stored at −20°C.

#### PCR amplification of target sequences

The amplification reactions were performed in 25 µl total volumes under complete aseptic conditions. Each PCR mixture contained 25-50 ng/µl genomic DNA, 10 pM/µl of each primers, 12.5 µl of ×2 Dream Taq Green PCR master mix (×2 buffer, 0.4 mMdNTP and 4 mM MgCl_2_; ThermoScientific, California, US), and 9 µl nuclease free water (Qiagen) to complete the total volume of the reactions. All amplifications were performed in a PTC-100™ thermal cycler (MJ Research Inc., USA) utilizing the following cycling profile; one cycle at 94°C for 5 min (initial denaturation) for all five markers. The PCR protocol of 18S rDNA, CO1, and 16S rDNA were amplified according to the following: 30 cycles denaturation at 94°C for 1min, annealing at 45°C for 1 min and elongation at 72°C for 1 min, and the final elongation at 72°C for 10 min [49,54]. While the ITS2 protocol included 30 cycles of denaturation at 94°C for 1.5 min, annealing at 55°C for 1.5 min and elongation at 72°C for 2 min, then the final elongation at 72°C for 10 min [50]. In addition to 12S rDNA was 5 cycles of denaturation at 94°C for 15 s, annealing at 51°C for 30 s and elongation at 68°C for 30 s, followed by 25 cycles denaturation at 94°C for 15 s, annealing at 53°C for 30 s and elongation at 70°C for 30 s, and the final elongation at 70°C for 5 min [57]. A reagent blank was run as control simultaneously with every PCR. Amplification success rate was measured based on the proportion of samples that produced sequences of the appropriate length. These sequences were further evaluated for their utility in species identification.

The PCR products were inspected by electrophoresis in 1.5% agarose gel in TBE buffer (45 mM Tris-borate, 1 mM EDTA, pH 8.3, Sigma-Aldrich) and stained with ethidium bromide (Sigma-Aldrich). A gene ruler 100 bp plus DNA ladder (Thermo Scientific, California, USA) was used with each gel. Gels photos were analyzed by Lab Image software (BioRad) [6].

### Phylogenetic analysis of amplified sequences

#### Sequencing of the obtained PCR products

PCR products were purified for sequencing using ExoSAP-IT PCR Product Cleanup Kit (Affymetrix, Ohio, USA) according to manufacturer’s recommendation. Sequencing reactions were performed in an MJ Research PTC-225 Peltier Thermal Cycler using an ABI PRISM^®^BigDye^™^ Terminator Cycle Sequencing Kits with AmpliTaq^®^ DNA polymerase (FS enzyme; Applied Biosystems), following the protocols supplied by the manufacturer. Each sequencing reaction was repeated at least three times in both the forward and reverse directions before being accepted for analysis [6]. The utilized primers sets for PCRs were used for sequencing of designated amplicons (Table-1).

#### Data submission in GenBank

The sequences of 18S rDNA, ITS2, 12S rDNA, CO1, and 16S rDNA of each tick species were aligned assembled and corrected using ChromasPro 1.49 beta (Technelysium Pty. Ltd., Tewantin, QLD, Australia), then the corrected ticks’ sequences were submitted in GenBank (http://www.ncbi.nlm.nih.gov/genbank/) to record each sequence with accession number.

#### Tree construction

Amplified sequences of each fragment were aligned using BLASTN program of NCBI (http://www.ncbi.nlm.nih.gov/BLAST/) for sequence homology searches against ticks’GenBank database. Multiple sequences alignments for evolutionary relationships between new Egyptian records and other ticks reference isolates were inferred using the ClustalW 1.8^®^ program [63] after modification of sequences length by BioEdit sequence alignmenteditor (v. 7.0.9.0). Two phylogenetic trees were constructed with the neighbor-joining method (NJ) [64], and the unweighted pair group method with arithmetic mean (UPGMA) [65]. The evolutionary distances were calculated by the maximum composite likelihood method [66]. Branches corresponding to partitions reproduced in less than 50% of bootstrap replicates were collapsed. The percentage of replicate trees in which the associated taxa clustered together in the bootstrap test (100 replicates) is shown next to the branches [67,68]. Phylogenetic analyses were conducted in MEGA4 [69].

## Results

### Prevalence of ticks and tick-borne Rickettsiae on dogs and camels

The tick-borne Rickettsiae infections rates were calculated in all tick species collected from camels and dogs in the three Egyptian provinces investigated. Hemolymph staining of tick specimens by Gimenez stain was successful for preliminary detection of Rickettsiae in ticks estimating the prevalence rate 11.89% in dog ticks and 10.1% in camel ticks (Table-2).

**Table-2:** The prevalence of Rickettsiae in ixodid tick species infesting dogs and camels by hemolymph staining technique with Gimenez stain and PCR using *OmpA* and *gltA* genes.

Ticks species	Tick number	Prevalence of tick species		Prevalence of Rickettsiae	

				Hemolymph staining	PCR using ompA and gltA genes

		Camels %	Dogs %	N (%)	N (%)
*R. sanguineus*	597/597	0	100	71/597 (11.89)	4/71 (5.6)
*Hayalomma* spp.	99/99	100	0	10/99 (10.1)	1/10 (10)
*H. dromedarii*	91/99	91.9	0	8/91 (8.79)	0 (0)
*H. marginatum*	5/99	5.05	0	1/5 (20)	1/1 (100)
*H. excavatum*	1/99	1.01	0	0 (0)	0 (0)
*H. impeltatum*	1/99	1.01	0	1/1 (100)	0 (0)
*H. rufipes*	1/99	1.01	0	0 (0)	0 (0)
Total	696	14.22	85.78	81/696 (11.6)	5/81 (6.17)

*H. dromedarii=Hyalomma dromedarii, R. sanguineus=Rhipicephalus sanguineus, H. marginatum=Hyalomma marginatum, H. excavatum=Hyalomma excavatum, H. impeltatum=Hyalomma impeltatum, H. rufipes=Hyalomma rufipes*, PCR=Polymerase chain reactions

**Figure-1:**
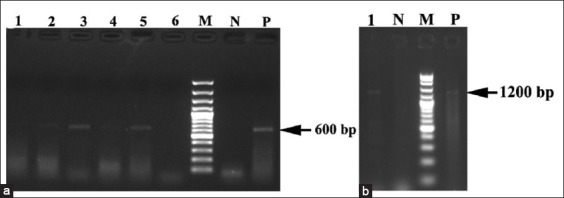
Molecular identification of tick-borne Rickettsiae by polymerase chain reaction products of the *OmpA* (a) and *gltA* (b) genes detected in *Rhipicephalus sanguineus* and *Hyalomma dromedarii* species in 1.5% agarose gels stained with ethiduim bromide. Lane M: 100 bp DNA ladder, Lane N: Control negative, Lane P: Control positive and Lane 1 and 6 are negative tick samples, while tick samples on Lanes 2 to 5 are *OmpA* positive (a) with molecular sized ranged from 600 bp. Whereas, Lane 1 (b) are *gltA* positive with molecular size 1200 bp.

Ticks which were positive by hemolymph staining technique were screened molecularly by *OmpA* and *gltA* genes with the fragment product 600 and 1200 bp, respectively (Figure-1). Five ticks were positive with *Rickettsia* spp. four of which obtained from *R. sanguineus* from Cairo province and one from *H. marginatum* from Sinai province. The prevalence rate of *Rickettsia* spp. was 6.17% depending on molecular identification of tick specimens by *OmpA* and *gltA* genes amplification (Table-2).

The six collected tick species from Cairo, Giza, and Sinai provinces were identified according to the developmental stage into adults and nymph; furthermore the adults were classified according to sex into females and males (Table-3). The brown dog tick *R. sanguineus* is the unique tick species that was found on dogs recording 100% infestation (Table-2). However, the camels tick *H. dromedarii* was the most abundant tick species found on camels recording 91.9% (Table-2). Lower percentages of infestation of other tick species were also recorded on camels such as *H. marginatum*, *H. impeltatum*, *H. excavatum* and *H. rufipes* (Table-2).

**Table-3:** The prevalence of tick species collected from camels and dogs in three provinces of Egypt.

Governorates	Localities of collection	Ticks species	Ticks fauna				

			♂	♀	Nymph	No.	Prevalence (%)
Cairo	3	3	276	353	9	638	
	El-Abbasia	*R. sanguineous*	257	338	4	599	93.88
	El-Dowaika	*R. sanguineous*	0	2	0	2	0.313
	Gisr El-Swiss	*H. dromedarii*	19	11	5	35	5.49
		*H. marginatum*	0	2	0	2	0.313
Giza	1	4	5	7	0	12	
	EL Haram	*H. dromedarii*	5	4	0	9	75
		*H. marginatum*	0	1	0	1	8.33
		*H. excavatum*	0	1	0	1	8.33
		*H. rufipes*	0	1	0	1	8.33
Sinai	1	3	38	17	0	55	
	Raas Sidr	*H. dromedarii*	37	15	0	52	94.55
		*H. marginatum*	1	1	0	2	3.64
		*H. impeltatum*	0	1	0	1	1.82

*H. dromedarii=Hyalomma dromedarii, R. sanguineus=Rhipicephalus sanguineus, H. marginatum=Hyalomma marginatum, H. excavatum=Hyalomma excavatum, H. impeltatum=Hyalomma impeltatum, H. rufipes=Hyalomma rufipes*.

### Morphological identification

**Figure-2:**
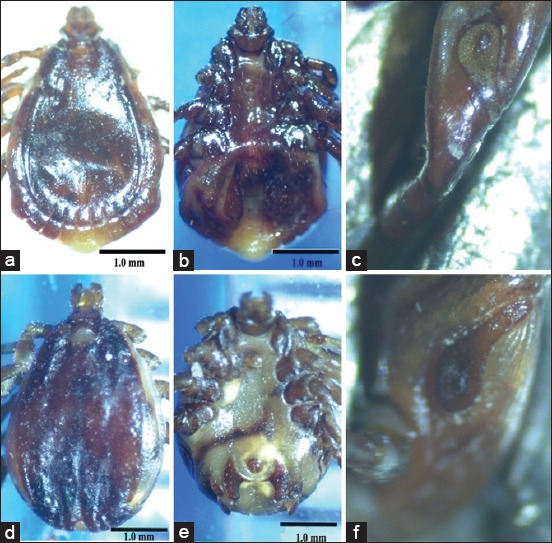
The adult males of the brown dog tick *Rhipicephalus sanguineus* and the camel tick *Hyalomma dromedarii* photographed by light microscope: (a) Dorsal view of *R. sanguineus*, (b) ventral view of *R. sanguineus*, (c) spiracular plate of *R. sanguineus*, (d) dorsal view of *H. dromedarii*, (e) ventral view of *H. dromedarii*, (f) spiracular plate of *H. dromedarii*.

**Figure-3:**
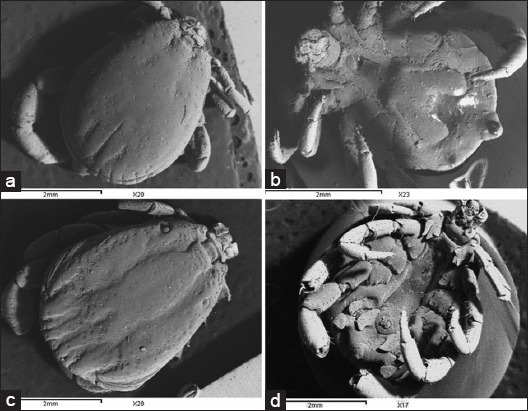
The adult males of the brown dog tick *Rhipicephalus sanguineus* and the camel tick *Hyalomma dromedarii* photographed by scanning electron microscope: (a) Dorsal view of *R. sanguineus*, (b) ventral view of *R. sanguineus*, (c) dorsal view of *H. dromedarii*, (d) ventral view of *H. dromedarii*.

**Figure-4:**
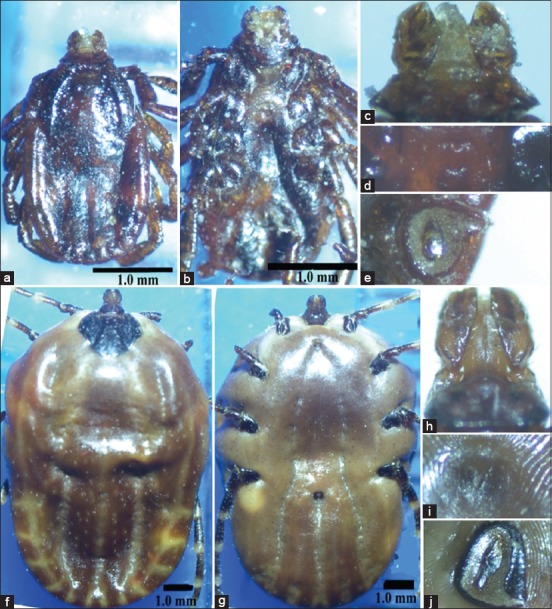
The adult females of the brown dog tick *Rhipicephalus sanguineus* and the camel tick *Hyalomma dromedarii* photographed by light microscope: (a) Dorsal view of *R. sanguineus*; (b) ventral view of *R. sanguineus*; (c) gnathosoma of *R. sanguineus*; (d) genital opening of *R. sanguineus*; (e) spiracular plate of *R. sanguineus*; (f) dorsal view of *H. dromedarii*; (g) ventral view of *H. dromedarii*; (h) gnathosoma of *H. dromedarii*; (i) genital opening of *H. dromedarii*; (j) spiracular plate of *H. dromedarii*.

**Figure-5:**
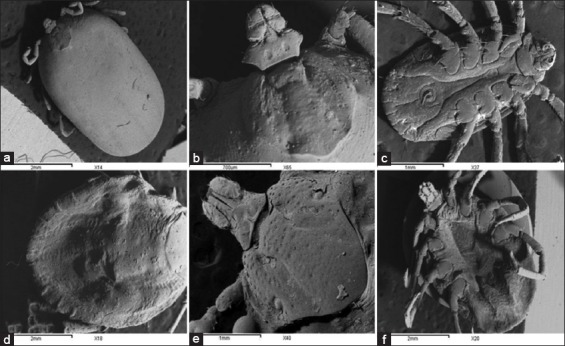
The adult females of the brown dog tick *Rhipicephalus sanguineus* and the camel tick *Hyalomma dromedarii* photographed by scanning electron microscope: (a and b) Dorsal view of *R. sanguineus*; (c) ventral view of *R. sanguineus*; (d and e) dorsal view of *H. dromedarii*; (f) ventral view of *H. dromedarii*.

Both male and female specimens which classified into *R. sanguineus* and *H. dromedarii* were photographed by LM and SEM for morphological description (Figures-2-5).

#### Males

LM showed that the color of the two tick species is similar (dark brown), while the size is different (*H. dromedarii* is larger than *R. sanguineus*). Additional characteristics are illustrated obviously by the LM such as grooves on the scutum, parma, adanal shields, subanal shields, and spiracular plates (Figure-2a-f). Lateral grooves are deep, narrow, and long (extending from eyes to the festoons) in *R. sanguineus*. They are deep and short (limited to the posterior third of the scutum) in *H. dromedarii*. Parma has a white color in both tick species while it protrudes beyond the festoons in *R. sanguineus* and at the level of festoons in *H. dromedarii*. Adanal shields have subtriangular shape rounded posteriorly in *R. sanguineus*. These shields have long margins strongly curved in *H. dromedarii*. Subanal shields are absent in *R. sanguineus* and present exteriorly of the axis of the adanal shields in *H. dromedarii*. Spiracular plates have long tails which are narrow less than the width of the adjacent festoon in *R. sanguineus*. In *H. dromedarii*, spiracular plates resemble those in *R. sanguineus* but their tails may be wider. The most previous characteristics in males of the two tick species were confirmed by SEM. Additional features were well distinguished by this instrument as posteromedian groove, paramedian grooves, caudal area of the scutum, and scutal punctuations (Figure-3a-d). The posteromedian groove is deep in both *R. sanguineus* and *H. dromedarii* but it is wider in *H. dromedarii*. The paramedian grooves are equal and shorter than posteromedian groove in the two tick species, while they are wider in *H. dromedarii* than *R. sanguineus*. In general, the caudal area of the scutum is more depressed in *H. dromedarii*. Scutal punctuations consist of four irregular rows of large punctuations in *R. sanguineus* and few scattered large punctuations in *H. dromedarii*.

#### Females

Most features in females of the two tick species can be distinguished by LM such as color, size, gnathosoma (mouth parts), genital aperture, and spiracular plates (Figure-4a-j). Some of these features are clearer with SEM beside additional characteristics as the outline of scutum and its punctuations (Figure-5a-f). The color of females in the two tick species is similar (dark brown) resembling males. The size of *H. dromedarii* female is larger than that of *R. sanguineus*. The dorsal view of gnathosoma in Figure-4c and h represents females and males in the two tick species. The dorsal surface of gnathosoma consists of basis capitulum and palpi. Basis capitulum has hexagonal shape with sharper lateral angles in *R. sanguineus* and tetragonal shape with blunt angles in *H. dromedarii*. Palpi are longer in *H. dromedarii* than in *R. sanguineus*. Genital aperture has broad U-shape in *R. sanguineus* and V-shape which in profile slopes gradually in *H. dromedarii*. Spiracular plates have small narrow tails in the two tick species, but the tails are slightly curved in *R. sanguineus*. Posterolateral margins of scutum are undulated in *R. sanguineus* and slightly convex in *H. dromedarii*. Scutum has large punctuations which scattered in *R. sanguineus* and denser over the central field and scapular fields in *H. dromedarii*.

### Molecular identification of tick genome by DNA markers

**Figure-6:**
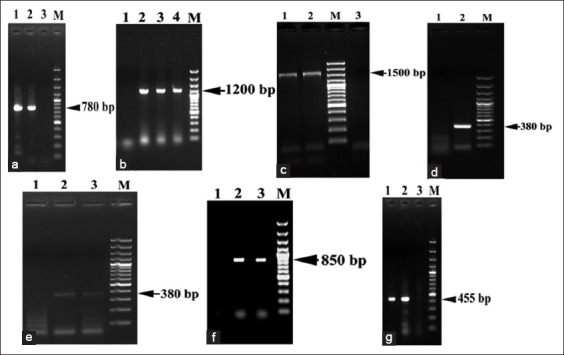
Molecular identification of tick species (*Rhipicephalus sanguineus* and *Hyalomma dromedarii*) by polymerase chain reaction products of the five DNA markers detected in 1.5% agarose gels stained with ethiduim bromide. In all figures Lane M: 100 bp DNA ladder, Lane 1 or 3: Control negative. (a) Lane 1 present 780 bp amplicon of 18S rRNA gene of *R. sanguineus*, while Lane 2 present 780 bp amplicon of 18S ribosomal ribonucleic acid (rRNA) gene of *H. dromedarii*, (b) 1200 bp amplicons of Second Internal transcribed spacer (ITS2) of *R. sanguineus*, (c) 1500 bp amplicons of ITS2 of *H. dromedarii*, (d and e) 380 bp amplicon of 12S rRNA gene of both *R. sanguineus* and *H. dromedarii*, (f) Lane 2 present 850 bp amplicon of cytochrome c oxidase subunit-1 (CO1) gene of *R. sanguineus*, while Lane 3 present 850 bp amplicon of CO1 gene of *H. dromedarii*, (g) Lane 1 present 455 bp amplicon of 16S rRNA gene of *R. sanguineus*, while Lane 2 present 455 bp amplicon of 16S rRNA gene of *H. dromedarii*.

*R. sanguineus* and *H. dromedarii* were screened by PCR using five specific primers of DNA markers; 18S rDNA, ITS2, 12S rDNA, CO1 and 16S rDNA, in accordance, the documented length of amplifications fragments in both tick species of 18S rDNA, 12S rDNA, CO1, and 16S rDNA were 780, 380, 850, 455 bp, respectively, while in ITS2 it was 1200 bp in *R. sanguineus* and 1500 bp in *H. dromedarii* (Figure-6a-g).

### Sequences analyses

The obtained sequences of the two tick species (*R. sanguineus* and *H. dromedarii*) were submitted in GenBank. The length of the obtained sequences varied from 355 to 1500 bp. Accession numbers of the sequences of the Egyptian tick amplified genes and other details as tick species, tick sexes, and localities of collection are listed in Table-4. Moreover, the identities of the Egyptian obtained sequences to other reference tick strains were ranged from 97% to 100% (Table-4). The partial sequence of 18S rDNA of *R. sanguineus* showed 99% identity with *R. sanguineus* (KF958435.1), while 18S rDNA of *H. dromedarii* showed 100% similarity to *H. dromedarii* (L76348.1). The identities of ITS2 were 99% and 96% of *R. sanguineus* and *H. dromedarii* obtained with reference strains *R. sanguineus* (JQ625707.1) and *H. dromedarii* (JQ733570.1), respectively. The sequence identities of partial sequence of 12S rDNA of *R. sanguineus* and *H. dromedarii* were 99% and 97% compared to *R. sanguineus* (JQ480844.1) and *H. dromedarii* (U95874.1). The similarity was 98-99% of CO1 gene of *R. sanguineus* and *H. dromedarii* compared with reference strains *R. sanguineus* (KM494916.1) and *H. dromedarii* (KM235697.1), respectively. Finally, the partial sequence of 16S rDNA of *R. sanguineus* showed 99% identity with *R. sanguineus* (KR870984.1), while 16S rDNA of *H. dromedarii* showed 98% similarity to *H. dromedarii* (L34306.1).

**Table-4:** GenBank accession numbers of sequenced genes were amplified from genomes of *R. sanguineus* and *H. dromedarii* and their identities with other reference strains.

DNA markers	Ticks		Governorates of ticks specimens	GenBank No.	Similarity with recorded tick species in GenBank		
	
	Species	Sex			Identity (%)	Covering (%)	Reference strains of tick species
18S rRNA	*R. sanguineous*	♀	Cairo	KU198407	99	93	KF958435.1
	*H. dromedarii*	♀	Giza, Sinai	KU198408	100	99	L76348.1
ITs2	*R. sanguineous*	♀	Cairo	KU198406	99	100	JQ625707.1
	*H. dromedarii*	♀	Giza, Sinai	KU214593	96	100	JQ733570.1
12S rRNA	*R. sanguineous*	♂	Cairo	KU198403	99	98	JQ480844.1
	*H. dromedarii*	♂	Cairo	KU963224	97	85	U95874.1
CO1	*R. sanguineous*	♂	Cairo	KU214592	98	99	KM494916.1
	*H. dromedarii*	♀	Giza, Sinai	KU323789	99	75	KM235697.1
16S rRNA	*R. sanguineous*	♀	Cairo	KU198404	99	99	KR870984.1
	*H. dromedarii*	♀	Giza, Sinai	KU198405	98	99	L34306.1

KF958435.1=*R. sanguineus* isolate 39_S_C4 18S rRNA gene, partial sequence, L76348.1=*H. dromedarii* 18S rRNA gene, JQ625707.1=*R. sanguineus* isolate 3464 5.8S ribosomal RNA gene, partial sequence; ITS2, complete sequence; and 28S rRNA gene, partial sequence, JQ733570.1=*H. dromedarii* 5.8S ribosomal RNA gene, partial sequence; ITS2, complete sequence; and 28S rRNA gene, partial sequence, JQ480844.1=*R. sanguineus* isolate 252_12s_SP6 12S rRNA gene, partial sequence; mitochondrial, U95874.1=*H. dromedarii* isolate Hydr6 12S rRNA gene, partial sequence; mitochondrial, KM494916.1=*R. sanguineus* isolate Gilan-e Gharb CO1 gene, complete cds; mitochondrial, KM235697.1=*H. dromedarii* voucher INHM: TC1314 CO1 gene, partial cds; mitochondrial, KR870984.1=*R. sanguineus* isolate Orkun-RS314 small subunit ribosomal RNA gene, partial sequence; mitochondrial, L34306.1=Mitochondrion *H. dromedarii* 16S rRNA gene. *H. dromedarii=Hyalomma dromedarii, R. sanguineus=Rhipicephalus sanguineus*, CO1=Cytochrome oxidase subunit-1, 12S rRNA=12S ribosomal ribonucleic acid, ITS2=Internal transcribed spacer 2, 28S rRNA=28S ribosomal ribonucleic acid

### Phylogenetic analysis

**Figure-7:**
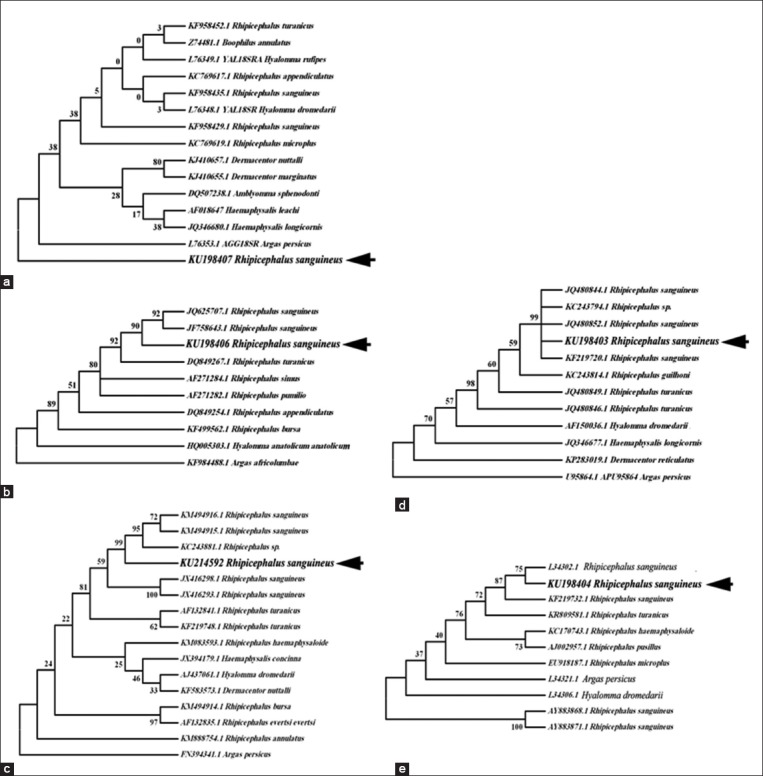
Phylogenetic trees of *Rhipicephalus sanguineus* based on the five DNA markers. All sequences of each marker were aligned and Neighbor-joining trees were constructed. (a) 18S rDNA, (b) Second internal transcribed spacer, (c) Cytochrome c oxidase subunit-1, (d) 12S rDNA, (e)16S rDNA.

**Figure-8:**
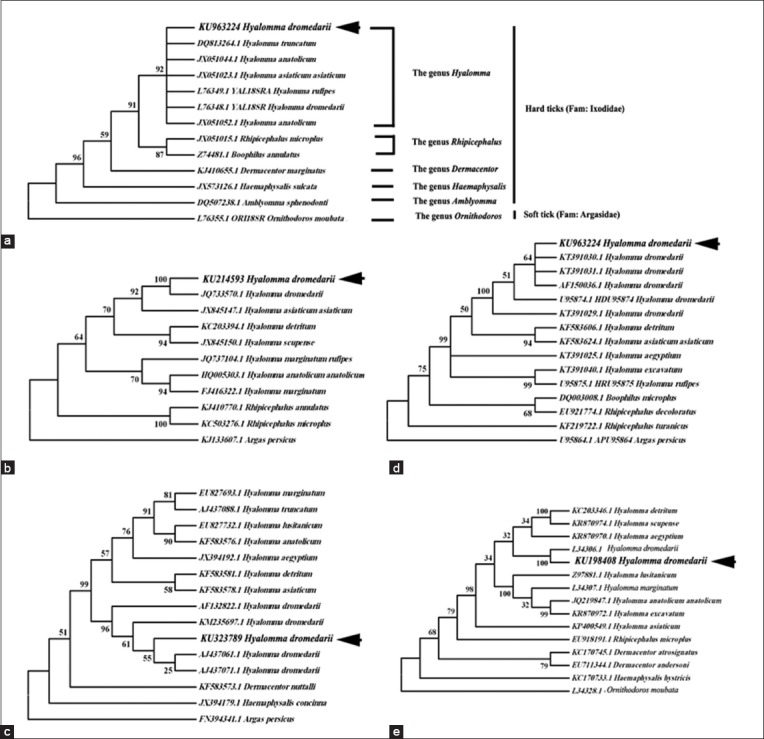
Phylogenetic trees of *Hyalomma dromedarii* based on the five DNA markers. All sequences of each marker were aligned and Neighbor-joining trees were constructed. (a) 18S rDNA, (b) Second internal transcribed spacer, (c) Cytochrome c oxidase subunit-1, (d) 12S rDNA, (e) 16S rDNA.

The phylogenetic tree of the two tick species (*R. sanguineus* and *H. dromedarii*) was constructed for each marker (Five DNA markers) based on Clustal W multiple alignments using two methods UPGMA and NJ method. Therefore, the only NJ method was discussed (Figures-7a-e and 8a-e). The phylogenitical analyses of five DNA markers of the two tick species sequences (*R. sanguineus* and *H. dromedarii*) were agreed with their morphological identification. The NJ trees of *R. sanguineus* were performed based on alignment of five DNA markers sequences using *Argas* spp. as outgroup inferred that *R. sanguineus* strains of this study were branched out or clustered together with *R. sanguineus* reference strains (Table-4) with a bootstrap value ranged from 87 to 99 (Figure-7a-e), except 18S rDNA tree *R. sanguineus* deposit in separate clade (Figure-7a). In *H. dromedarii*, the NJ trees were constructed based on alignment of five DNA markers sequences using *Argas persicus* or *Ornithodoros* spp.(soft ticks) as outgroup indicated that *H. dromedarii* strains of this study in all five markers were branched out or clustered together with *H. dromedarii* reference strains (Table-4) with a bootstrap value ranged from 51 to 100 (Figure-8a-e).

## Discussion

Nowadays, the studies on emerging and reemerging infectious diseases as Ricketsioses are increased due to their public health implication, zoonotic importance in addition to, increasing the contact between wild and domestic animals, animals and human, as well as animals, human and vectors [11,12,70-72]. So that the proper diagnostic tools, such as molecular techniques, are needed to improve the sensitivity and specificity of the diagnosis of Rickettsiae. Therefore, this study was designed to achieve two goals. The first goal is to determine the ability of ixodid tick species infesting dogs and camels to carry Rickettsiae using Gimenez stain and PCR. The second goal is to illustrate the accurate taxonomic status of the two most common ticks, the brown dog tick *R. sanguineus*, and the camel tick *H. dromedarii* using a combination of LM, SEM and molecular analysis.

Hemolymph staining technique of collected ticks revealed that the prevalence rate of *Rickettsia* spp. was 11.89% in dog ticks and 10.1% in camel ticks (Table-2). The total prevalence rate in ticks was 11.6% (Table-2). While the prevalence rate of *Rickettsia* spp. depending on molecular identification of tick specimens by *OmpA* and *gltA* genes amplification was 6.17% (Table-2 and Figure-1). The low prevalence rate of Rickettsiae in ticks in this study may retain to the low numbers of *Rickettsia* spp. circulated in the blood of the host even during the acute phase of the disease [73,74]. Furthermore, the *Rickettsia* DNA in the host blood and their vectors (ticks) can be detected only during rickettsemia (recent infection) [75]. Moreover, the infection rates of *Rickettsia* spp. in ticks under natural condition were tend to be low (<1%) due to the lethal effect of Rickettsiae on ticks [76,77].

In Egypt, there were few reports documented the presence of *Rickettsia* spp. in *R. sanguineus* [5], while many studies in the surrounding countries had detected *Rickettsia* spp. in *R. sanguineus* as Algeria, Tunisia and Israel [78-81]. Moreover, the previous reports detected the presence *R. aeschlimannii*, *R. africae*, and other *Rickettsia* spp. by PCR in *H. dromedarii* from Egypt [3,5,6] and in other adjacent countries as Tunisia, Algeria, Israel and the United Arab Emirates [82-86]. Subsequently, this work confirms the role of both tick spp. (*R. sanguineus* and *H. dromedarii*) in transmission of *Rickettsia* spp. in Egypt and the importance of domestic dogs and camels as potential infection amplifiers. This finding agree with Piranda *et al*. [87] and Kamani *et al*. [88] who found Rickettsiae in both dogs and camels.

The brown dog tick *R. sanguineus* is the unique tick species that was found on dogs recording 100% infestation (Tables-2 and 3). The result confirms the fact that *R. sanguineus* is still the main dog tick in Egypt which agrees with previous reports [28,30]. In camels, *H. dromedarii* was the most abundant tick species found on camels recording 91.9%, meanwhile lower percentages of infestation of other tick species were also recorded such as *H. marginatum*, *H. impeltatum*, *H. excavatum*, and *H. rufipes* (Table-2). Others studies were in agreement with our results as the most dominant tick species on camels are *H. dromedarii* [28,32,89]. The taxonomic features in males and females of both tick species can be differentiated by LM but some of these features show clearer with SEM. The most dominant differential feature between the two ticks’ species is the basis capitulum of gnathosoma which hexagonal in shape with sharp lateral angles in *R. sanguineus* while tetragonal in shape with blunt angles in *H. dromedarii*. Furthermore, palpi are shorter in *R. sanguineus* than in *H. dromedarii* (Figures-4c, h and 5b, e). Moreover, the lateral grooves in *R. sanguineus* are deep, narrow and long while deep and short in *H. dromedarii* (Figures-2a, d and 3a, c). *R. sanguineus* adanal plates have subtriangular shape rounded posteriorly, but in *H. dromedarii* have long margins strongly curved and subanal plates are absent in *R. sanguineus* and present exteriorly of the axis of the adanal plates in *H. dromedarii* (Figures-2b, e and 3b, d). In addition to, genital aperture of *R. sanguineus* female has broad U-shape but, V-shape which in profile slopes gradually in *H. dromedarii* female (Figures-4d, i and 5c, f). These results agree with the taxonomic keys depending on the morphological characterization of [28,29,41,42,90].

Morphological similarities at both intra- and inter-species level limit the usefulness of morphological taxonomic key such as in *R. sanguineus* group [45,46,91]. However, molecular taxonomy will become standard approach for tick taxonomy confirming morphological identification. Moreover, the presented phylogenetic analyses in this study were pioneer ones, hence, no earlier report of similar molecular taxonomy and evolutionary analyses based on the five DNA markers in Egypt.

In this study, the utilized five DNA markers; nuclear (18S rDNA and ITS2) and mitochondrial (12S rDNA, CO1 and 16S rDNA), revealed that the documented length of amplified fragments of 18S rDNA, 12S rDNA, CO1 and 16S rDNA were 780, 380, 850, and 455 bp, respectively, are the same in length within both tick species. While ITS2 was different 1200 bp in *R. sanguineus* and 1500 bp in *H. dromedarii* (Figure-6a-g).

In *R. sanguineus*, phylogenetic trees based on the five DNA markers showed that the Egyptian ticks species (KU198407, KU198406, KU198403, KU214592 and KU198404) were close to the reference counterparts (KF958435.1, JQ625707.1, JQ480844.1, KM494916.1 and KR870984.1) which obtained from the surrounding geographical countries as Israel, Iran and Turkey (the temperate strain group) (Figure-7a-e). These results agree with Dantas-Torres *et al*. [42], Moraes-Filho *et al*. [92], Sanches *et al*. [93] who divided the *R. sanguineus* group into tropical and temperate strains. On the other hand, the phylogenetic tree of 18S rDNA gene sequence (KU198407), placed the Egyptian brown dog tick in a separate clad that may return to 18S rRNA identified at genera level (KF958435.1) [51], subsequently, doubted the usefulness and applicability of such gene in molecular taxonomy of ticks (Figure-7a). In *H. dromedarii*, the evolutionary relationship is more clear, sharp, and easier to be interpreted since the phylogenetic trees based on the five DNA markers (KU198408, KU214593, KU963224, KU323789 and KU198405) placed the Egyptian species together with the reference ones (L76348.1, JQ733570.1, U95874.1, KM235697.1 and L34306.1) derived from different geographical countries as Iraq, Israel, India and America (Figure-8a-e).

On the other hand, it is obvious that mitochondrial markers have several advantages over the nuclear genes during this study. The sequence length of both 12S rDNA and 16S rDNA are <450 bp, even the 850 bp length of CO1 can be obtained in one reaction in contrast to the length of 18S rDNA and ITS2 fragments. Moreover, the evolutionary rate and sequence alignment of mitochondrial genes is faster than that calculated for nuclear genes [94,95]. In addition to, the reference database of complete and partial mitochondrial genomes of several tick species are more available than nuclear genes [44,54-57,95-97]. This is return to the fact that mitochondrial genes have strict and simple maternal inheritance compared to nuclear DNA [98].

## Conclusion

The detection of Rickettsiae in ixodid tick species and establishment of the pylogenetic status of *R. sanguineus* and *H. dromedarii* would be useful in studying the geographical distribution and understanding the epidemiology of ticks and tick-borne Rickettsioses in Egypt which consequently help in the control strategy against the disease. Moreover, this study confirms the suitability of the mitochondrial genes as DNA markers for reliable identification of ticks at both intra and interspecies level over the nuclear ones.

## Authors’ Contributions

AE, FAS, NATA, AAG and SA designed and supervised the experiments. HHAMA collected tick samples from dogs and camels. All authors shared in protocols of staining technique, LM, SEM, PCR, sequences, GenBank submission and phylogentetic trees. All authors contributed in draft and revision of the manuscript. All authors read and approved the final manuscript.

## References

[1] Soliman A.K, Botros B.A, Ksiazek T.G (1989). Seroprevalence of *Rickettsia typhi* and *Rickettsia conorii* infection among rodents and dogs in Egypt. J. Trop. Med. Hyg.

[2] Corwin A, Habib M, Olson J (1992). The prevalence of arboviral, rickettsial, and Hantaan-like viral antibody among schoolchildren in the Nile River Delta of Egypt. Trans. R. Soc. Trop. Med. Hyg.

[3] Lange J.V, El Dessouky A.G, Manor E (1992). Spotted fever Rickettsiae in ticks from the Northern Sinai Governate, Egypt. Am. J. Trop. Med. Hyg.

[4] Socolovschi C, Barbarot S, Lefebvre M, Parola P, Raoult D (2010). *Rickettsia sibirica mongolitimonae* in traveler from Egypt. Emerg. Infect. Dis.

[5] Loftis A.D, Reeves W.K, Szumlas D.E (2006). Rickettsial agents in Egyptian ticks collected from domestic animals. Exp. Appl. Acarol.

[6] Abdel-Shafy S, Allam A.T.N, Mediannikov O, Parola P, Raoult D (2012). Molecular detection of spotted fever group Rickettsiae associated with ixodid ticks in Egypt. Vector Borne Zoonotic Dis.

[7] Parola P, Raoult D (2001). Ticks and tick borne bacterial diseases in human: An emerging infectious threat. Clin. Infect. Dis.

[8] Parola P, Davoust B, Raoult D (2005). Tick-and flea-borne rickettsial emerging zoonoses. Vet. Res.

[9] Dantas-Torres F, Chomel B.B, Otranto D (2012). Ticks and tick-borne diseases: A one health perspective. Trends Parasitol.

[10] Kernif T, Socolovschi C, Bitam I, Raoult D, Parola P (2012). Vector-borne rickettsioses in North Africa. Infect. Dis. North Am.

[11] Wieten R.W, Hovius J.W, Groen E.J, van der Wal A.C, de Vries P.J, Beersma M.F, Tijsse-Klasen E, Sprong H, Grobusch M.P (2011). Molecular diagnostics of *Rickettsia Africae* infection in travelers returning from South Africa to The Netherlands. Vector Borne Zoonotic Dis.

[12] Gargili A, Palomar A.M, Midilli K, Portillo A, Kar S, Oteo A.J (2012). *Rickettsia* species in ticks removed from humans in Istanbul, Turkey. Vector Borne Zoonotic Dis.

[13] Parola P, Paddock D.C, Socolovschi C, Labruna B.M, Mediannikov O, Kernif T, Abdad Y.M, Stenos J, Bitam I, Fournier P, Raoult D (2013). Update on tick-borne Rickettsioses around the world: A geographic approach. Clin. Microbiol. Rev.

[14] Gimenez D.F (1964). Staining *Rickettsiae* in yolk sac cultures. Stain. Technol.

[15] Fournier P.E, Raoult D, Raoult D, Parola P (2007). Bacteriology, taxonomy, and phylogeny of *Rickettsia*. Rickettsial Diseases.

[16] Kang Y.J, Diao X.N, Zha G.Y, Chen M.H, Xiong Y, Shi M, Fu W.M, Guo Y.J, Pan B, Chen X.P, Holmes E.C, Gillespie J.J, Dumler S.J, Zhang Y.Z (2014). Extensive diversity of Rickettsiales bacteria in two species of ticks from China and the evolution of the Rickettsiales. BMC Evol. Biol.

[17] Raoult D, Roux V (1997). Rickettsioses as paradigms of new or emerging infectious diseases. Clin. Microbiol. Rev.

[18] Anderson J.F, Magnorelli L.A (2008). Biology of ticks. Infect. Dis. Clin. North Am.

[19] Socolovschi C, Mediannikov O, Raoult D, Parola P (2009). The relationship between spotted fever group Rickettsiae and ixodid ticks. Vet. Res.

[20] Hechemy K.E, Avsic-Zupanc T, Childs E.J, Raoultd A.D (2003). Rickettsiology: Present and future directions - Preface. Ann. N. Y. Acad. Sci.

[21] Dumler J.S, Barbet F.A, Bekker P.J.C, Dasch A.G, Palmer H.G, Ray C.S, Rikihisa Y, Rurangirwa R.F (2001). Reorganization of genera in the families Rickettsiaceae and Anaplasmataceae in the order Rickettsiales: Unification of some species of *Ehrlichia* with *Anaplasma*, *Cowdria* with *Ehrlichia* and *Ehrlichia* with *Neorickettsia*, descriptions of six new species combinations and designation of *Ehrlichia equi* and ‘HGE agent’ as subjective synonyms of *Ehrlichia phagocytophila*. Int. J. Syst. Evol. Microbiol.

[22] Fournier P.E, Dumler J.S, Greub G, Zhang J, Wu Y, Raoult D (2003). Gene sequence-based criteria for identification of new rickettsia isolates and description of *Rickettsia heilongjiangensis* sp. Nov. J. Clin. Microbiol.

[23] Roux V, Fournier P.E, Raoult D (1996). Differentiation of spotted fever group rickettsiae by sequencing and analysis of restriction fragment length polymorphism of PCR-amplified DNA of the gene encoding the protein rOmpA. J. Clin. Microbiol.

[24] Roux V, Rydkina E, Eremeeva M, Raoult D (1997). Citrate synthase gene comparison, a new tool for phylogenetic analysis, and its application for the Rickettsiae. Int. J. Syst. Bacteriol.

[25] Fournier P.E, Roux V, Raoult D (1998). Phylogenetic analysis of spotted fever group rickettsiae by study of the outer surface protein rOmpA. Int. J. Syst. Bacteriol.

[26] Mediannikov O.Y, Sidelnikov Y, Ivanov L (2004). Acute tick-borne rickettsiosis caused by *Rickettsia heilongjiangensis* in Russian Far East. Emerg. Infect. Dis.

[27] Guillemi C.E, Tomassone L, Farber D.M (2015). Tick-borne Rickettsiales: Molecular for the study of an emergent group of pathogens. J. Micrbiol. Methods.

[28] Hoogstraal H, Kaiser M.N (1958). The ticks (Ixodoidea) of Egypt: A brief review and keys. J. Egypt Public Health Assoc.

[29] Estrada-Pena A, Bouattour A, Camicas J.L, Walker A.R (2004). Ticks of Domestic Animals in the Mediterranean Region: A Guide to Identification of Species.

[30] Haridy F.M, Hassan A.A, Hafez A.O, El-Sherbini G.T, Morsy T.A (2009). External and intestinal parasites of pet dogs with reference to zoonotic toxocariasis. J. Egypt. Soc. Parasitol.

[31] Abdel-Shafy S (2000). Microbiological and control studies on ticks infesting farm animals and poultry. PhD Thesis.

[32] El-Kammah K.M, Oyoun L.M, El Kady G.A, Abdel-Shafy S (2001). Investigation of blood parasites in livestock infested with argasid and ixodid ticks in Egypt. J. Egypt. Soc. Parasitol.

[33] Abdel-Shafy S, Allam N.A.T (2013). Quantitative real-time RT-PCR detection of flaviviruses associated with camel ticks in Egypt. Glob. Vet.

[34] Qiu W.G, Dykhuizen D.E, Acosta M.S, Luft B.J (2002). Geographic uniformity of the lyme diseases spirochete (*borrelia burgdorferi*) and its shared history with tick vector (ixodes scapularis) in the northeastern United States. Genetics.

[35] Mazyad S.A, Khalaf S.A (2002). Studies on Theileria and *Babesia* infecting live and slaughtered animals in Al Arish and El Hasanah, North Sinai Governorate, Egypt. J. Egypt. Soc. Parasitol.

[36] Mazyad S.A, Hafez A.O (2007). Q fever (*Coxiella burnetii*) among man and farm animals in North Sinai, Egypt. J. Egypt. Soc. Parasitol.

[37] Abdel-Shafy S (2008). Scanning electron microscopy and comparative morphology of *Hyalomma anatolicum excavatum H. dromedarii* and *H. marginatum marginatum* (Acari: Ixodidae) based on nymphs. Acarologiya.

[38] Caporale D.A, Rich S.M, Spielman A, Telford S.R, Kocher T.D (1995). Discriminating between Ixodes ticks by means of mitochondrial DNA sequences. Mol. Phylogenet. Evol.

[39] Guglielmone A.A, Venzal J.M, Gonzáleh Z, Acuña D, Nava S, Hinojosa A, Mangold A.J (2006). The phylogenetic position of Ixodes stilesi Neumann 1911 (Acari: Ixodidae): Morphological and preliminary molecular evidences from 16S rDNA sequences. Syst. Parasitol.

[40] Barker S.C, Murrell A (2004). Systematics and evolution of ticks with list of valid genus and species names. Parasitology.

[41] Chen Z, Yang X, Bu F, Liu J (2010). Ticks (Acari: Ixodoidae: Argasidae, Ixodidae) of China. Exp. Appl. Acarol.

[42] Dantas-Torres F, Latrofa S.M, Annoscia G, Giannelli A, Parisi A, Otranto D (2013). Morphological and genetic diversity of *Rhipicephalus sanguineus sensu lato* from the new and old worlds. Parasit. Vectors.

[43] Gray J, Dantas-Torres F, Estrada-Pena A, Levin M (2013). Systematics and ecology of the brown dog tick, *Rhipicephalus sanguineus*. Ticks Tick Borne Dis.

[44] Lui G.H, Chen Y.Z, Song H.Q, Lin R.Q, Zhou D.H, Zhu X.Q (2013). Complete mitochondrial genome sequance data provides evidence that dog tick *Rhipicephalus sanguineus* (Acari: Ixodidae) represents a species complex. Int. J. Biol. Sci.

[45] Guglielmone A.A, Robbins R.G, Apanaskevich D.A, Petnery T.N, Estrada-Pena A (2014). The Hard Ticks of the World, (Acari: Ixodidae).

[46] Nava S, Estrada-Pena A, Petney T, Beati L, Labruna B.M, Szabo P.J.M, Venzal M.J, Mastropaolo M, Mangold J.A, Guglielmone A.A (2015). The taxonomic status of Rhipicephalus sanguineus (Latreille, 1806). Vet. Parasitol.

[47] Chen X, Yu Z, Guo l, Li L, Meng H, Wang D, Liu R, Liu J (2012). Life cycle of *haemaphysalis doenitzi* (Acari: Ixodidae) under laboratoty condition and its phylogeny based on mitochondrial 16s rDNA. Exp. Appl. Acarol.

[48] Nava S, Mastropaolo M, Venzal J.M, Mangold A.J, Guglielmone A.A (2012). Mitochondrial DNA analysis of *Rhipicephalus sanguineus* sensu lato (Acari: Ixodidae) in the Southern Cone of South America. Vet. Parasitol.

[49] Lv J, Wu S, Zhang Y, Zhang T, Feng C, Jia G, Lin X (2014). Development of a DNA barcoding system for the Ixodida (Acari: Ixodida). Mitochond. DNA.

[50] Lv J, Wu S, Zhang Y, Chen Y, Feng C, Yuan X, Jia G, Deng J, Wang C, Wang Q, Mei L, Lin X (2014). Assessment of four DNA fragments (CO1, 16S rDNA, ITS2, 12S rDNA) for species identification of the Ixodida (Acari: Ixodida). Parasit. Vectors.

[51] Dobson S.J, Barker S.C (1999). Phylogeny of the hard ticks (Ixodidae) inferred from 18S rRNA indicates that the genus *Aponomma* is paraphyletic. Mol. Ecol. Resour.

[52] Mangold A.J, Bargues M.D, Mas-Coma S (1998). Mitochondrial 16S rDNA sequences and phylogenetic relationships of species of *Rhipicephaus* and other tick genera among Metastriata (Acari: Ixodidae). Parasitol. Res.

[53] Norris D.E, Klompen J.S.H, Black W.C (1999). Comparison of the mitochondrial 12s and 16s ribosomal DNA genes in resolving phylogenetic relationships among hard ticks (Acari: Ixodidae). Ann. Entomol. Soc. Am.

[54] Chitimia L, Lin R, Cosoroaba I, Wu X, Song H, Yuan Z, Zhu X (2010). Genetic characterization of ticks from Southwestern Romania by sequences of mitochondrial cox1 and nad5 genes. Exp. Appl. Acarol.

[55] Song S, Shao R, Atwell R, Barker S, Vankan D (2011). Phylogenetic and phylogeographic relationships in *Ixodes holocyclus* and *Ixodes cornuatus* (Acari: Ixodidae) inferred from COX1 and ITS2 sequences. Int. J. Parasitol.

[56] Erster O, Roth A, Wolkomirsky R, Leibovich B, Shkap V (2013). Comparative analysis of mitochondrial markers from four species of *Rhipicephalus* (Acari: Ixodidae). Vet. Parasitol.

[57] Beati L, Keirans J.E (2001). Analysis of the systematic relationships among ticks of the genera *Rhipicephalus* and *Boophilus* (Acari: Ixodidae) based on mitochondrial 12S ribosomal DNA gene sequences and morphological characters. J. Parasitol.

[58] Abdullah S, Helps C, Tasker S, Newbury H, Wall R (2016). Ticks infesting domestic dogs in the UK: A large-scale surveillance programme. Parasit. Vectors.

[59] Burgdorfer W (1970). Hemolymph test, a technique for detection of Rickettsiae in ticks. Am. J. Trop. Med. Hyg.

[60] Regnery R.L, Spruill C.L, Plikaytis D (1991). Genotypic identification of rickettsiae and estimation of intraspecies sequence divergence for portions of two Rickettsial genes. J. Bacteriol.

[61] Zilberman N, Reikhav S, Hulata G, Ron M (2006). High-throughput genomic DNA extraction protocol from Tilapiaʼs fine tissue. Aquaculture.

[62] Sambrook J, Fritsch E.F, Maniatis T (1989). Molecular Cloning. A Laboratory Manual.

[63] Dessen P, Fondrat C, Valencien C, Mugnier C (1990). BISANCE: A French service for access to biomolecular databases. CABIOS.

[64] Saitou N, Nei M (1987). The neighbor-joining method: A new method for sequences. J. Mol. Biol.

[65] Dawyndt P, Demeyer H, De Baets B (2006). UPGMA clustering revisited: A weight-driven approach to transitive approximation. Int. J. Approx. Reason.

[66] Tamura K, Nei M, Kumar S (2004). Prospects for inferring very large phylogenies by using the neighbor-joining method. Proc. Natl. Acad. Sci. USA.

[67] Felsenstein J (1985). Confidence limits on phylogenies: An approach using the bootstrap. Evolution.

[68] Felsenstein J (2004). Inferring Phylogenies. 1.

[69] Tamura K, Dudley J, Nei M, Kumar S (2007). MEGA4: Molecular evolutionary genetics analysis (MEGA) software version 4.0. Mol. Biol. Evol.

[70] Keysary A, Eremeeva M.E, Leitner M, Din A.B, Wikswo E.M, Mumcuoglu Y.k, Inbar M, Wallach D.A, Shanas U, King R, Waner T (2011). Spotted fever group rickettsiae in ticks collected from wild animals in Israel. Am. J. Trop. Med. Hyg.

[71] Socolovschi C, Reynaud P, Raoult D, Parola P (2011). Rickettsiae and Borrelia in ticks on migratory birds from the Camargue National Park, France.

[72] Movila A, Alekseev A.N, Dubinina H.V, Toderas I (2012). Detection of tick-borne pathogens in ticks from migratory birds in the Baltic region of Russia. Med. Vet. Entomol.

[73] Breitschwerdt E.B, Levy M.G, Davidson M.G, Walker D.H, Burgdorfer W, Curtis B.C, Babineau C.A (1990). Kinetics of IgM and IgG responses to experimental and naturally acquired *Rickettsia rickettsii* infection in dogs. Am. J. Vet. Res.

[74] Parola P, Paddock C.D, Raoult D (2005). Tick-borne rickettsioses around the world: Emerging diseases challenging old concepts. Clin. Microbiol. Rev.

[75] Horta M.C, Labruna M.B, Pinter A, Linardi P.M, Schumaker T.T (2007). *Rickettsia* infection in five areas of the state of Sao Paulo, Brazil. Mem. Inst. Oswal Cruz.

[76] Levin M.L, Killmaster L, Eremeeva M.E, Dasch G.A (2009). Effects of *Rickettsia conorii* infection on the survival of *Rhipicephalus sanguineus* ticks. Clin. Microbiol. Infect.

[77] Socolovschi C, Matsumoto K, Brouqui P, Raoult D, Parola P (2009). Experimental infection of *Rhipicephalus sanguineus* with *Rickettsia conorii conorii*. Clin. Microbiol. Infect.

[78] Harrus S, Perlman-Avrahami A, Mumcuoglu K.Y, Morick D.G, Baneth G (2011). Molecular detection of *Rickettsia massiliae, Rickettsia sibiricamongolitimonae* and *Rickettsia conorii israelensis* in ticks from Israel. Clin. Microbiol. Infect.

[79] Znazen A, Hammami B, Lahiani D, Ben Jemaa M, Hammami A (2011). Israeli spotted fever, Tunisia. Emerg. Infect. Dis.

[80] Lalzar I, Harrus S, Mumcuoglu Y.K, Gottlieb Y (2012). Composition and seasonal variation of *Rhipicephalus turanicus* and *Rhipicephalus sanguineus* bacterial communities. Appl. Environ. Microbiol.

[81] Leulmi H, Aouadi A, Bitam I, Bessas A, Benakhla A, Raoult D, Parola P (2016). Detection of *Bartonella tamiae*, *Coxiella burnetii* and rickettsiae in arthropods and tissues from wild and domestic animals in northeastern Algeria. Parasit. Vectors.

[82] Demoncheaux J.P, Socolovschi C, Davoust B, Haddad S, Raoult D, Parola P (2012). First detection of *Rickettsia aeschlimannii* in *Hyalomma dromedarii* ticks from Tunisia. Ticks Tick Borne Dis.

[83] Djerbouh A, Kernif T, Beneldjouzi A, Socolovschi C, Kechemir N, Parola P, Raoult D, Bitam I (2012). The first molecular detection of *Rickettsia aeschlimannii* in the ticks of camels from Southern Algeria. Ticks Tick Borne Dis.

[84] Kernif T, Djerbouh A, Mediannikov O, Ayach B, Rolain J.M, Raoult D, Parola P, Bitam I (2012). *Rickettsia africae* in *Hyalomma dromedarii* ticks from sub-Saharan Algeria. Ticks and Tick-borne Dis.

[85] Kleinerman G, Baneth G, Mumcuoglu K.Y, Van Straten M, Berlin D, Apanaskevich D.A, Abdeen Z, Nasereddin A, Harrus S (2013). Molecular detection of *Rickettsia africae*, *Rickettsia aeschlimannii*, and *Rickettsia sibirica mongolitimonae* in camels and *Hyalomma* spp. ticks from Israel. Vector-Borne Zoonot. Dis.

[86] Al-Deeb M.A, Muzaffar S.B, Abu-Zeid Y.A, Enan M.R, Karim S (2015). First record of a spotted fever group *Rickettsia* sp. and *Theileria annulata* in *Hyalomma dromedarii* (Acari: Ixodidae) ticks in the United Arab Emirates. Florida Entomol.

[87] Piranda E.M, Faccini J.L.H, Pinter A, Pacheco R.C, Cancado P.H.D, Labruna M.B (2011). Experimental infection of *Rhipicephalus sanguineus* ticks with the bacterium *Rickettsia rickettsii*, using experimental infected dogs. Vector Borne Zoonotic Dis.

[88] Kamani J, Baneth G, Apanaskevich D.A, Mumcuoglu K.Y, Harrus S (2015). Molecular detection of *Rickettsia aeschlimannii* in *Hyalomma* spp. ticks from camels (*Camelus dromedarius*) in Nigeria, West Africa. Med. Vet. Entomol.

[89] Fard S.R, Fathi S, Asl E.N, Nazhad H.A, Kazeroni S.S (2012). Hard ticks on one humped camel (Camelus dromedarius) and their seasonal population dynamics in southeast, Iran. Trop. Anim. Health Prod.

[90] Pegram R.G, Clifford C.M, Walker J.B, Keirans J.E (1987). Clarification of the *Rhipicephalus sanguineus* group (Acari, Ixodoidea, Ixodidae). I. *R. sulcatus* Neumann, 1908 and *R. turanicus* Pomerantsev, 1936. Syst. Parasitol.

[91] Walker J.B, Keirans J.E, Horak I.G (2000). The Genus *Rhipicephalus* (Acari, Ixodidae): A Guide to the Brown Ticks of the World.

[92] Moraes-Filho J, Marcili A, Nieri-Bastos F.A, Richtzenhain L.J, Labruna M.B (2011). Genetic analysis of ticks belonging to the *Rhipicephalus sanguineus* group in Latin America. Acta Trop.

[93] Sanches G.S, Évora P.M, Mangold A.J, Jittapalapong S, Rodriguez-Mallon A, Guzmán P.E, Bechara G.H, Camargo-Mathias M.I (2016). Molecular, biological, and morphometric comparisons between different geographical populations of *Rhipicephalus sanguineus sensu lato* (Acari: Ixodidae). Vet. Parasitol.

[94] Navajas M, Fenton B (2000). The application of molecular markers in the study of diversity in acarology: A review. Exp. Appl. Acarol.

[95] Shao R, Barker S (2007). Mitochondrial genomes of parasitic arthropods: Implications for studies of population genetics and evolution. Parasitology.

[96] Dergousoff S.J, Chilton N.B (2007). Differentiation of three species of ixodid tick, *Dermacentor andersoni*, *D. variabilis* and *D. albipictus*, by PCR-based approaches using markers in ribosomal DNA. Mol. Cell Probes.

[97] Latrofa S.M, Dantas-Torres F, Annoscia G, Cantacessi C, Otranto D (2013). Comparative analyses of mitochondrial and nuclear genetic markers for the molecular identification of *Rhipicephalus* spp. Infect. Genet. Evol.

[98] White D.J, Wolff J.N, Pierson M, Gemmell N.J (2008). Revealing the hidden complexities of mtDNA inheritance. Mol. Ecol.

